# Lung Cancer, Myocardial Infarction, and the Grossarth-Maticek Personality Types: A Case-control Study in Fukuoka, Japan

**DOI:** 10.2188/jea.11.281

**Published:** 2007-11-30

**Authors:** Jun Nagano, Nobuyuki Sudo, Chiharu Kubo, Suminori Kono

**Affiliations:** 1 Department of Preventive Medicine, Kyushu University Graduate School of Medical Sciences.; 2 Institute of Health Science, Kyushu University.; 3 Department of Psychosomatic Medicine, Kyushu University Graduate School of Medical Sciences.

**Keywords:** lung cancer, myocardial infarction, personality, Grossarth-Maticek, case-control study

## Abstract

Grossarth-Maticek and colleagues have shown, in their prospective studies, a strong relationship of their personality types, Types 1 and 2, to cancer and coronary heart disease (CHD), respectively. Relevant information is limited from replication studies, and little is known about psychosocial factors in relation to cancer or CHD in Japan. Subjects included 95 cases of lung cancer (LC), 94 cases of myocardial infarction (MI) and 596 controls. The controls were men and women who visited a clinic for a health checkup. The Grossarth-Maticek personality types, Types 1 to 6, were assessed using the Short Interpersonal Reactions Inventory. The distributions of the 6 personality types were compared between the case and control groups, adjusting for sex and age class. The relation of *each* of the 6 types to LC and MI were examined in terms of odds ratio, using a logistic regression model controlling for age, sex, job status, education level, and smoking status. As regards the distribution analysis, Types 1 and 2 in the LC and MI groups each were not more prevalent than the controls, respectively. High score of the Type 1 scale was associated with a statistically nonsignificant decrease in LC risk. MI risk was significantly, positively associated with the Type 2 and 5 scales, and unexpectedly, positively related to the Type 3 scale. The present findings partly supported the Grossarth-Maticek theory, but there remain some conflicting issues to be confirmed in future studies.

## INTRODUCTION

Much interest has drawn to the role for psychosocial factors in the etiology of cancer and coronary heart disease (CHD)^1^^, ^^2^^)^. For example, Type A behavior pattern, characterized by competition, hostility, impatience, and exaggerated commitment to work, has been reported to be related to an increased risk of CHD in white collar white men^3^^)^ and people who lack social support^4^^, ^^5^^)^. More recently, hostility, a major attribute of Type A behavior pattern, has been regarded as a potential “toxic” element in this personality construct^2^^, ^^6^^)^. An increased risk of cancer has been linked to the loss-depression-hopelessness syndrome and the lack of emotional reaction or its suppression^7^^)^. A recent review of prospective studies found relatively consistent results for the latter trait^1^^)^, which is also called Type C behavior pattern^8^^)^.

In the mid 1960s, Grossarth-Maticek and his colleagues^9^^)^ interviewed 1,353 healthy inhabitants of a small town in Yugoslavia, and classified them into one of 4 personality types, Types 1 to 4, on the basis of his theory of disease-prone personalities; Type 1 was defined as a tendency to react to stress with feelings of hopelessness and depression; Type 2 as a personality of reacting to stress with feelings of anger, hostility, and aggression; Type 3 tended to show an ambivalent reaction between those typical of Types 1 and 2; and Type 4 was defined as an autonomous, healthy type. Types 1, 2, and 4 would share psychosocial constructs and their theoretical disease-proneness with Type C, Type A/hostility, and Type B, respectively, while Type 3 is rather unique and difficult to place in well-known concepts of disease-prone personalities^9^^)^. In a 10 year follow-up period, Types 1 and 2 were strongly, positively associated with the risk of cancer and CHD/apoplexy, respectively, as compared with Types 3 and 4 each^9^^)^. These findings were reproduced in two other cohort studies in Germany^9^^)^. In addition, intervention studies showed that “autonomy training”, a cognitive behavioral therapy to change the behavioral patterns from Types 1 and 2 to Type 4, lowered the subsequent risk of cancer and CHD^10^^, ^^11^^)^. Later, the Grossarth-Maticek stress-personality theory (GM-SPT) was revised by adding 2 types^12^^)^; Type 5 was constructed to represent a trait of ‘rational, anti-emotional reactions to stress’ which was associated with both cancer and CHD/apoplexy^13^^)^; and Type 6 was conceptualized as an antisocial, psychopathic type, that would possibly be criminal and prone to drug addiction, but not to cancer nor CHD. An English version of the Short Interpersonal Reactions Inventory (SIRI) was developed to measure the 6 personality types of GM-SPT^12^^)^.

Despite all the evidence from the series of studies by Grossarth-Maticek et al, not many studies have addressed the relation of these personality types to morbid events independently. To date, only limited information is available from retrospective studies in European countries^14^^-^^18^^)^, and their results are inconsistent. Little is known about psychosocial factors in relation to cancer or CHD in Japan^19^^-^^22^^)^. We addressed the question as to whether the GM-SPT was applicable to the Japanese population and, more specifically, whether Types 1 and 2 were associated with increased risks of cancer and CHD, respectively, in a case-control study of lung cancer (LC) and myocardial infarction (MI).

## MATERIALS AND METHODS

Study subjects included 95 patients with LC, 94 patients with acute or old MI, and 596 healthy people as controls. LC patients were a series of patients admitted to a hospital from February to December, 1998, and MI patients were a series of either in-patients or out-patients at two hospitals from February to September, 1998. Eligible cases were patients at the age of less than 70 who were not too severely ill to complete a self-administered questionnaire without assistance. Diagnoses of LC and MI were made by pulmonary oncologists and cardiologists, respectively, and all of the patients had been informed of their diagnoses before the present survey. Controls were those who underwent a health checkup of the Fukuoka Institute of Occupational Health from February to September, 1998 and who did not have a history of cancer, MI, or apoplexy. The reasons for the checkup were as follows: (1) annual checkup under a contract between the institute and the subject’s company, (2) voluntary individual-based participation with subsidy from city/town governments, or (3) voluntary individual-based participation without the subsidy. The controls were consecutively asked to participate in the study so that the numbers in sex- and age-specific categories (40–49, 50–59, and 60–69 years in each sex) were around 100; we recruited this numbers of controls in order to examine the SIRI by sex and age class^23^^)^. All the cases and controls were residents of Fukuoka City or neighboring municipalities, Japan.

The eligible subjects were asked to answer a set of self-administered questionnaires including a Japanese version of the SIRI^23^^)^, and received a book coupon (500 yen) as gratitude. Response rates, the number of participants divided by the number of people who were asked to participate in the study, were 94.1, 96.9, and 82.4% for the LC case group, MI case group, and control group, respectively. Table 1 shows the distribution of the subjects by sex- and age-class. In both LC and MI patients, women were fewer than men, and patients aged less than 50 years were fewer than those over 50 years. In the LC patients, 82% had been diagnosed within one year before the administration of questionnaires, and 76% were in a relatively advanced stage, *i.e.*, stage IIIa or more^24^^)^. On the other hand, of the MI patients, only 28% had been diagnosed within the previous year, more than half had a single vessel disease, and the majority had unlimited or mildly limited daily activity.

**Table 1.** Age and sex distribution of the control, lung cancer, and myocardial infarction groups.

Factor	Category	Control		Lung cancer		Myocardial infarction	
		
		N	%	N	%	N	%
Sex	Men	320	53.7	70	73.7	74	78.7
	Women	276	46.3	25	26.3	20	21.3
Age, y	< 50	219	36.7	17	17.9	8	8.5
	50–59	220	36.9	39	41.1	19	20.2
	60+	157	26.3	39	41.1	57	71.3
Total		601	100.0	95	100.0	94	100.0

The SIRI contains 70 items with a dichotomous answer, ‘yes’ or ‘no’^12^^)^. The inventory consists of 6 scales which correspond to the 6 Grossarth-Maticek personality types. Each scale consists of 10 items except for the Type 4 scale; Type 4 is represented by 20 items including 10 reverse items. The score for each scale is the number of positive responses, while the score for Type 4 is divided by two so that all scales score between 0 and 10. The reliability and validity of the Japanese version of the SIRI used in the present study have been examined by the authors^23^^)^. Cronbach alphas of the 6 scales from Types 1 to 6 were 0.71, 0.72, 0.56, 0.69, 0.65, and 0.49, respectively; and test-retest correlation coefficients were 0.83, 0.79, 0.64, 0.82, 0.76, and 0.56. A person was assigned to a type with the highest score of the 6; if she/he had equal scores for two or more types, she/he was assigned to the type with lower mean score in the controls; *i.e.*, Type 6 had the highest priority followed by Types 2, 3, 1, 5, and 4 in order. Lifestyle factors such as smoking status, alcohol drinking, education level, and working status were also determined by a self-administered questionnaire.

The distributions of the 6 personality types as determined among the LC and MI groups by the SIRI were each compared with the distribution in the control group, adjusting for sex and age class (< 55 and 55+ years old). The relation of *each* of the SIRI scales (Types 1 to 6) to LC and MI were examined in terms of odds ratio (OR). The subjects were classified into three categories according to the scores of a scale of interest so that numbers of controls in each category were as equal as possible. ORs with 95% confidence intervals (CI) for each category of the scale were estimated, and the corresponding trend was tested using an unconditional logistic regression model. This model included indicator variables representing age (< 50, 50-59, and 60+ years old), sex, job status (current worker, retiree, and housewife), and education level (junior high school, high school, and junior college or higher), because these factors were found to be related to the SIRI scales in the present control subjects^23^^)^; smoking status (never smoker, ever smoker, and missing information) was also included as a covariate. Because none of the scores of the SIRI subscales was significantly associated with either the reason for visiting the clinic in the control group or the difference in hospital in the MI group, each of the control and MI groups was treated as a single group. The analyses were done using the SAS software^25^^)^. Reported *p* values were two-sided, and *p* values less than 0.05 were regarded as statistically significant.

## RESULTS

Table 2 shows the distribution of the Grossarth-Maticek personality types in the control, LC, and MI groups by sex and age. Overall, the largest number of subjects were allocated to Type 4, followed by Types 5 and 1. Types 2 and 3 accounted for small percentages, and Type 6 was rare. The distribution pattern of the LC group was not significantly different from that of the control group. Type 1 was no more prevalent in the LC group than in the control group. The distribution of the MI group was significantly different from the control group, largely due to fewer Type 4 subjects and more Type 5 subjects in the MI group as compared with the control group. No difference was found in the proportion of Type 2 between the MI and control groups.

**Table 2.** Distribution of the Grossarth-Maticek personality types in the control, lung cancer (LC), and myocardial infarction (MI) groups by sex.

Sex and group	Total number	Number (%)												P-value^a^

		Type 1		Type 2		Type 3		Type 4		Type 5		Type 6		
Men														
Control	320	52	(16.3)	5	( 1.6)	11	(3.4)	180	(56.3)	72	(22.5)	0	(0.0)	
LC	70	12	(17.1)	3	( 4.3)	2	(2.9)	34	(48.6)	18	(25.7)	1	(1.4)	0.11 ^b^
MI	74	13	(17.6)	1	( 1.4)	1	(1.4)	27	(36.5)	32	(43.2)	0	(0.0)	0.04 ^b^
Women														
Control	276	65	(23.6)	16	( 5.8)	3	(1.1)	127	(46.0)	61	(22.1)	4	(1.5)	
LC	25	2	( 8.0)	1	( 4.0)	1	(4.0)	16	(64.0)	5	(20.0)	0	(0.0)	0.31 ^b^
MI	20	4	(20.0)	3	(15.0)	0	(0.0)	9	(45.0)	4	(20.0)	0	(0.0)	0.26 ^b^
Total														
Control	596	117	(19.6)	21	( 3.5)	14	(2.4)	307	(51.5)	133	(22.3)	4	(0.7)	
LC	95	14	(14.7)	4	( 4.2)	3	(3.2)	50	(52.6)	23	(24.2)	1	(1.1)	0.72 ^c^
MI	94	17	(18.1)	4	( 4.3)	1	(1.1)	36	(38.3)	36	(38.3)	0	(0.0)	0.04 ^c^

^a^ Based on chi-square for the general association.

^b^ Controlling for age class (<55 and 55+ years).

^c^ Controlling for sex and age class (<55 and 55+ years).

Table 3 shows the relation between the 6 SIRI scales and LC and MI risk. The highest level of Type 1 was marginally significantly associated with a *decreased* risk of LC, as compared with the lowest level. Types 2, 3, and 5 showed a clear, positive association with MI, and each trend was statistically significant. Type 4 was not related to LC risk, but had a modest, inverse association with MI risk, although the dose response was not significant.

**Table 3.** Odds ratio (OR) with 95% confidence interval (CI)^a^ of lung cancer and myocardial infarction for levels of the SIRI subscales.

Scale	Score	No. control	Myocardial infarction		Myocardial infarction	
	
			No. cases	OR (95% CI)	No. cases	OR (95% CI)
Type 1	0–3	194	38	1.00	33	1.00
	4–6	228	40	0.95 (0.55–1.62)	32	0.98 (0.54–1.77)
	7+	174	17	0.54 (0.28–1.05)	29	1.12 (0.60–2.08)
				*P* = 0.09 ^b^		*P* = 0.74 ^b^
Type 2	0	158	18	1.00	16	1.00
	1–2	248	45	1.58 (0.82–3.04)	35	1.42 (0.71–2.85)
	3+	188	32	1.61 (0.81–3.20)	43	2.40 (1.19–4.83)
				*P* = 0.22 ^b^		*P* = 0.01 ^b^
Type 3	0–1	173	24	1.00	13	1.00
	2–3	229	36	1.29 (0.69–2.38)	35	2.36 (1.14–4.92)
	4+	194	35	1.20 (0.65–2.21)	46	3.15 (1.54–6.49)
				*P* = 0.60 ^b^		*P* = 0.002 ^b^
Type 4	0–6	165	26	1.00	29	1.00
	6.5–8	279	41	0.89 (0.49–1.61)	44	0.77 (0.43–1.39)
	8.5+	152	28	1.07 (0.56–2.07)	21	0.64 (0.32–1.28)
				*P* = 0.81 ^b^		*P* = 0.20 ^b^
Type 5	0–5	207	30	1.00	18	1.00
	6–7	207	38	1.27 (0.71–2.27)	33	1.35 (0.68–2.67)
	8+	182	27	1.00 (0.53–1.90)	43	1.99 (1.02–3.90)
				*P* = 0.99 ^b^		*P* = 0.04 ^b^
Type 6	0	243	31	1.00	32	1.00
	1	220	32	1.05 (0.59–1.86)	32	0.89 (0.49–1.60)
	2+	131	32	1.31 (0.71–2.40)	30	1.15 (0.61–2.16)
				*P* = 0.40 ^b^		*P* = 0.71 ^b^

^a^ Adjusted for age, sex, job status, education level, and smoking status, using a logistic regression model.

^b^ Test for a linear trend.

Because the strong, positive association of Type 3 with MI was an unexpected finding, we examined the items for Type 3 in detail. The relatively low alpha coefficient (0.56) suggested that Type 3 may have consisted of heterogeneous items. The items representing Type 3 were loaded on by 3 factors identified by a factor analysis of the SIRI^23^^)^. Six of 10 items for Type 3 were loaded on more than 0.4 by one factor and less than 0.3 by the others. These items are shown in Table 4. Three sets of 2 items could be interpreted as representing ambivalent attitude towards an object (ambivalence), egocentric tendency (egoism), and impatience in seeking for fulfillment of one’s needs by others (impatience). We thus examined the relation between MI risk and each of these 3 components. As shown in Table 5, impatience was significantly, positively associated with MI risk, while the other two components, ambivalence and egoism, were not clearly related to the risk.

**Table 4.** Components of the Type 3 construct and corresponding items.

*Ambivalence*	
10.	I alternate to a great degree between the positive and negative evaluation of people and conditions.
17.	With people I love, I keep changing from keeping them at a great distance to stifling dependence, and from stifling dependence to excessive distancing.
*Egoism*	
3.	I am mainly concerned with my own wellbeing.
45.	I seek satisfaction of my own needs and desires first, regardless of the needs and rights of others.
*Impatience*	
24.	When I am in a situation which I experience as threatening, I immediately try to get other people to help and support me.
52.	When I make emotional demands on another person, I require immediate satisfaction.

**Table 5.** Odds ratio (OR) with 95% confidence interval (CI)^a^ of myocardial infarction for levels of components included in the Type 3 construct.

Component	Score	No. control	No. cases	OR (95% CI)
Ambivalence	0	485	74	1.00
	1–2	108	20	1.01 (0.57–1.98)

Egoism	0	191	20	1.00
	1	156	25	1.17 (0.58–2.36)
	2	247	49	1.34 (0.72–2.52)
				*P* = 0.35 ^b^
Impatience	0	246	28	1.00
	1	217	44	2.82 (1.56–5.10)
	2	131	22	2.68 (1.31–5.46)
				*P* = 0.002 ^b^

^a^ Adjusted for age, sex, job status, education level, and smoking status, using a logistic regression model.

^b^ Test for a linear trend.

## DISCUSSION

We reported here a case-control study to examine applicability of the GM-SPT in a Japanese population. The distribution of the 6 Grossarth-Maticek personality types was not shown to have as clear an association with LC or with MI as has been demonstrated in the series of studies by Grossarth-Maticek and his colleagues^9^^, ^^12^^)^. However, the present study showed a nearly significant, inverse association between Type 1 and LC in contrast to the Grossarth-Maticek studies. The findings that Types 2 and 5 were associated with MI are consistent with the GM-SPT. Type 3 was unexpectedly, strongly associated with an increased risk of MI.

The LC and MI cases were out-patients and/or in-patients at 3 hospitals in the study area. The controls were those who took a health checkup, and they were not necessarily representatives of residents in the study area. In this regard, the comparability between cases and controls may not have been guaranteed. However, we adjusted for sex, age, education level, job status, and smoking habits which may have influenced the process of selecting cases and controls. Because the controls included those who voluntarily took a health checkup, it may be argued that these people might have been more anxious than the general population. This may have affected the disease-personality relationships. However, as noted in the Material and Methods section, the SIRI scales did not differ according to the reasons for taking a checkup^23^^)^.

Methodological problems need to be clarified in interpreting the present findings. As pointed out elsewhere^1^^, ^^26^^)^, results from studies addressing a psychosocial issue as a potential cause of cancer are tenuous when they are of a retrospective design, and they are difficult to interpret; the observed difference in characteristics between cancer patients and controls may be due to the impact of the cancer diagnosis. This concern may be especially relevant to the LC patients in the present study; all of them had been informed of their illness, and some 80% were at relatively advanced stages. The impact of diagnosis may also have altered the psychological characteristics of MI patients to some extent. Yet such an impact might be smaller in MI patients than in LC patients, because the survival of MI patients is much better than that of LC patients in Japan^27^^)^ and is more favorable in those who have survived the acute period of the disease^28^^)^. The observed Type 2–MI association cannot be ascribed to a possible general distress in patients caused by having a serious illness, since such an association was not seen for LC.

Failure to find the Type 1–cancer association observed by Grossarth-Maticek and his colleagues might be related to another weakness of the present study–the method of questionnaire-administration. Grossarth-Maticek and Eysenck admitted that their original inventory was difficult and intricate in its wording (p. 302)^29^^)^, and the inventory required subjects complex introspection and self-revelation^30^^, ^^31^^)^. Their questionnaire-administration was always assisted by specially trained interviewers, who tried to establish trust and explained each item as well as their notion of the stress/personality–disease relationship. In an experimental subcohort of their study, they used a simple handing-out method without an interviewer’s assistance and found that psychosocial measures were much less predictive of subsequent disease incidence^30^^, ^^31^^)^. When developing the Japanese version of the SIRI, we asked several groups of people, including patients with cancer and CHD, how they understood the questions, and rephrased the items so that they could be understood as easily as possible^23^^)^. Consequently, the present SIRI subscales, at least for Types 1, 2, 4, and 5 had a satisfactory retest-reliability. However, as Grossarth-Maticek pointed out^30^^)^, the problem of self-revelation is closely linked to the hypothesis that a major aspect of the cancer-prone personality is the suppression of feelings and emotional responses, and such denial may lead to differential responding in different conditions of test administration. Non-expression of negative emotion might have affected such an association with MI to a lesser degree than with LC.

Direct information from independent studies that examined the *distribution* of the 6 Grossarth-Maticek personality types in relation to cancer or CHD are very limited. No study used the SIRI to address this issue. Using the Personality Stress Inventory, a longer version of the SIRI developed by Grossarth-Maticek^12^^)^, Schmitz interviewed 100 persons who attended a relaxation program and classified them into the 6 personality types; 6 of 7 persons with ‘cancerous or precancerous’ diagnosis during the last 5 years were allocated to Type 1, and 4 of 8 with MI diagnosis were allocated to Type 2^15^^)^. Instead of the original Grossarth-Maticek method, he assigned a subject to a given type if any one score fell into the highest quartile and the other scores did not. This method has, however, been criticized because the method failed to assign more than two thirds of the subjects to any one of the 6 types^32^^)^.

Amelang and his colleagues constructed 6 ‘revised scales’ corresponding to the Grossarth-Maticek types, from a pool of items in several of Grossarth-Maticek’s original questionnaires^33^^)^. In 2 separate case-control studies using the revised scales in Germany, the Type 1 scale failed to discriminate people reported to have cancer diagnosis from healthy subjects^17^^)^. Another study in Germany supported the GM-SPT in a *quasi-prospective* design^14^^)^. Using 10 questions selected from those developed by Grossarth-Maticek for predicting cancer, Quander-Blaznik^14^^)^ interviewed patients visiting a clinic for treatment of respiratory symptoms *before* diagnosis was made. The author found that patients later diagnosed as having LC had scored significantly higher on the scales such as ‘low expression of anxiety’ and ‘unfulfilled need for closeness’, traits which are components of Type 1^9^^, ^^34^^)^. In the present study, Type 1 showed an *inverse* association with LC risk. This may be due to some unknown, but considerable, cultural difference between Western countries and Japan; a behavioral pattern leading to distress in one cultural condition might lead to no distress or even to well-being in another.

An MI/CHD case-control study in Norway found no significant difference in the Type 2 score of the SIRI between 58 MI patients and 70 randomly selected control subjects^16^^)^. In another population-based, mailed questionnaire survey in Norway that identified 106 subjects with MI or a history of MI, the Type 2 score of the SIRI was not significantly associated with MI risk^18^^)^. A case-control study in Germany found no association between CHD and the Type 2 scale revised by Amelang et al^17^^)^. Contrary to these studies, the present case-control study showed that the Type 2 scale was significantly, positively associated with MI risk. The present finding support the validity of the SIRI Type 2 scale derived from the GM-SPT.

Direct information on the relationship between Type 5 and CHD is not so far available from cohort studies by Grossarth- Maticek and his colleagues. However, in their early prospective study in Yugoslavia, ‘rationality and anti-emotionality’, which corresponds to the Type 5 concept defined later^12^^)^, was shown to be positively associated with mortality from CHD and apoplexy as well as from cancer^13^^)^. Contrary to this finding, an *inverse* association was found between stroke and the rationality/anti-emotionality scale developed by Grossarth-Maticek et al^13^^)^ in a large-scale cross-sectional survey in Japan^35^^)^. The positive association between Type 5 and MI in the present study is compatible with the Grossarth-Maticek findings, although Type 5 was unrelated to LC.

The Type 3 scale was also significantly, positively related to MI. Interestingly, among the 3 components included in the Type 3 construct (ambivalence, egoism, and impatience), only impatience was significantly associated with an increased risk of MI. Ambivalence and egoism are core components of Type 3 in the GM-SPT, as described in the above studies as “ambivalence” (p. 480)^9^^)^ or “selfish-behavior” (p. 39)^34^^)^. On the other hand, impatience might be closely related not only to Type 3 but also to Type A behavior, a known CHD risk factor^26^^)^.

In summary, in a Japanese population, the original type-allocation method using the SIRI failed to replicate Grossarth-Maticek et al’s findings with regard to cancer and CHD proneness. However, an analysis examining the relationship of each SIRI scale with LC and MI found that Types 2 and 5 were positively associated with MI, partly supporting the GM-SPT. An unexpected, positive relation between Type 3 and MI and a modest, inverse association between Type 1 and LC need to be confirmed in future studies, preferably of a prospective design.
