# ERRATUM: Lung Cancer, Myocardial Infarction, and the Grossarth-Maticek Personality Types: a Case-control Study in Fukuoka, Japan

**DOI:** 10.2188/jea.12.188

**Published:** 2007-11-30

**Authors:** Jun Nagano, Nobuyuki Sudo, Chiharu Kubo, Suminori Kono

The fourth column in the first row of Table 3 (p. 284): “myocardial infraction” is to replaced by “lung cancer.” Corrected Table is as follows;

**Table 3.** Odds ratio (OR) with 95% confidence interval (CI)^a^ of lung cancer and myocardial infarction for levels of the SIRI subscales.

Scale	Score	No. control	Lung Cancer		Myocardial infarction	
	
			No. cases	OR (95% CI)	No. cases	OR (95% CI)
Type 1	0–3	194	38	1.00	33	1.00
	4–6	228	40	0.95 (0.55–1.62)	32	0.98 (0.54–1.77)
	7+	174	17	0.54 (0.28–1.05)	29	1.12 (0.60–2.08)
				*P* = 0.09 ^b^		*P* = 0.74 ^b^
Type 2	0	158	18	1.00	16	1.00
	1–2	248	45	1.58 (0.82–3.04)	35	1.42 (0.71–2.85)
	3+	188	32	1.61 (0.81–3.20)	43	2.40 (1.19–4.83)
				*P* = 0.22 ^b^		*P* = 0.01 ^b^
Type 3	0–1	173	24	1.00	13	1.00
	2–3	229	36	1.29 (0.69–2.38)	35	2.36 (1.14–4.92)
	4+	194	35	1.20 (0.65–2.21)	46	3.15 (1.54–6.49)
				*P* = 0.60 ^b^		*P* = 0.002 ^b^
Type 4	0–6	165	26	1.00	29	1.00
	6.5–8	279	41	0.89 (0.49–1.61)	44	0.77 (0.43–1.39)
	8.5+	152	28	1.07 (0.56–2.07)	21	0.64 (0.32–1.28)
				*P* = 0.81 ^b^		*P* = 0.20 ^b^
Type 5	0–5	207	30	1.00	18	1.00
	6–7	207	38	1.27 (0.71–2.27)	33	1.35 (0.68–2.67)
	8+	182	27	1.00 (0.53–1.90)	43	1.99 (1.02–3.90)
				*P* = 0.99 ^b^		*P* = 0.04 ^b^
Type 6	0	243	31	1.00	32	1.00
	1	220	32	1.05 (0.59–1.86)	32	0.89 (0.49–1.60)
	2+	131	32	1.31 (0.71–2.40)	30	1.15 (0.61–2.16)
				*P* = 0.40 ^b^		*P* = 0.71 ^b^

^a^ Adjusted for age, sex, job status, education level, and smoking status, using a logistic regression model.

^b^ Test for a linear trend.

## Instructions to Authors

JOURNAL OF EPIDEMIOLOGY is the official scientific Journal of the JAPAN EPIDEMIOLOGICAL ASSOCIATION. Manuscripts on human and experimental epidemiology are accepted from members of the Association in any country. In addition, contribution from a nonmember in foreign countries is also accepted without submission fees, but with reprints provided at actual cost. However, there will be no charge for a limited number of reprints to contributors from Asia and Pacific region.

There are five types of publication: 1) Articles reporting original research; 2) Reviews; 3) Statistical data; 4) Short communications (concisely worked up reports not exceeding two printed pages including figures and tables); 5) Letters to the Editor, which provide a forum for communication of opinion, interpretation and new information on various aspects of epidemiology. In addition, occasional reviews and editorials on current topics may be printed by invitation.

All materials submitted for publication, including solicited articles, are subject to editorial review and revision. Submission of a paper implies that it has been approved by all of the named authors, that data in support of all findings are retained by the authors, that the paper reports unpublished work, and that it is not simultaneously under consideration for publication elsewhere.

Accepted manuscripts become the property of the Japan Epidemiological Association and may not be published elsewhere in the same form, either in English or in any other language, without the consent of the Editorial Office.

### Submission of Manuscripts

Address manuscripts to:

Yosikazu Nakamura, M.D., M.P.H., F.F.P.H.M., *Journal of Epidemiology* Editorial Office, Department of Public Health, Jichi Medical School, 3311-1 Yakushiji, Minamikawachi, Tochigi 329-0498, Japan.

Telephone: 0285-58-7338 Facsimile: 0285-44-7217 E-mail: edit-je@jichi.ac.jp

### Manuscript Form

Manuscripts, written in English, should be submitted in triplicate, i.e., one original and two copies. Each copy of the manuscript should be accompanied by one complete set of illustrations (not photocopies). The entire manuscript including references, tables and figure legends, must be typed double-spaced with pica typescript (10 letters/2.5 cm) and with ample margins on all sides. Do not justify the right-hand margin. Material prepared on a word processor should be printed with a letter quality printer, if possible.

A floppy disc written by standard ASCII file could be accepted in addition to the typed manuscript to shorten the printing period. This is recommended when the manuscript contains many tables.

English Language. Authors are responsible for the linguistic accuracy of their papers. If not writing in his mother tongue, authors are advised to ask a subject specialist with a sound knowledge of English to check and, if necessary, correct his work. After acceptance, all manuscripts will be forwarded to the language editor for review, but this in no way diminishes the responsibility of the author to pay meticulous attention to the linguistic accuracy of the manuscript.

### Arrangement of Manuscript

The manuscript for regular papers should be divided into sections with appropriate section headings. The sequence of the manuscript should be as follows : Title page, Abstract/Key Words, Introduction, Materials and Methods, Results, Discussion, Acknowledgments (if any), References, Tables, and Figure legends.

In the upper right-hand corner of each page, type the first author’s last name plus page number (title page being page 1).

**Title Page.** The title page should contain the article title, author’s name (s), institutional affiliation (s), running title (not exceeding 40 letters including spaces), and the name and mailing address of the person to whom proofs, correspondence and reprint requests should be sent.

**Abstract.** The abstract should be a concise statement in no more than 200 words of 1) what was done, 2) what was found, and 3) what was concluded. Vague, general statements such as “The significance of the study is discussed,” or “The clinicoepidemiological aspects of this entity are summarized,” are uninformative and not acceptable. Below the abstract, provide three to five key words for cross-indexing.

**Introduction.** This section should contain a statement of the purpose or aim of the work, the problem that stimulated the work, and a brief summary of previous relevant investigations. It is not necessary to include all of the background literature in this section.

**Materials and Methods.** Materials and procedures must be presented in sufficient detail so that the work can be repeated by other investigators. Methods previously published should not be described in detail but merely cited in appropriate references. The sources of special reagents or instrumentation used in the study should be given along with the location of the manufacturer.

All units of measurement should be those in international use (preferably SI units). Temperatures should be given in degrees Centigrade. Omit periods after units of measurement (e.g., cm, mg, ml, hr, min, sec, 37C, etc.) and do not use plurals. Use % in the text, rather than per cent or percent.

Experiments involving either human subjects or materials of human origin should be carried out in accordance with the principles embodied in the Declaration of Helsinki of 1975. For animal experiments, authors should follow the guidelines for the care and use of laboratory animals. In the case of research involving recombinant DNA, experiments should be earned out according to the guidelines issued by the authorized agency in the country where the research is performed. Avoid detailed mathematical explanation, and these are summarized in the Annex.

**Results.** This section should contain a concise description of the data tables and figures should be used to allow easy comprehension of the data. Excessive verbal explanation of the data given in tables and figures should be avoided.

**Discussion.** In this section, the results should be interpreted and related to existing knowledge in the field. Information already presented in the introduction or results sections should not be repeated.

**Acknowledgments.** Financial support, aid in technical fields, statistical analyses, photography, stenography, advice from colleagues, etc. can be acknowledged.

**References.** The references should be numbered consecutively in the order that they are first mentioned in the text. (Note : references are not to be listed alphabetically.) The references may contain only published work and papers in press. Unpublished data, manuscripts submitted but not yet accepted, and personal communications are specifically excluded from the reference list. However, they may be indicated within the text in parentheses ; for example, (E. Saito, personal communication). Identify references in the text, tables and legends by Arabic numerals in parentheses. References should conform to the following style:

1. Tsugane S, Gotlieb SLD, Laurenti R, de Souza JMP, Watanabe S. Cancer mortality among Japanese residents of the city of Sao Paulo, Brazil. Int J Cancer, 1990 ; 45 : 436-439.
2. Gastl G, Ward JM, Rapp UR. Immunocytochemistry of oncogenes. In ; Polak JM and Van Noorden S, eds. Immunocytochemistry: Modern methods and applications, 2nd ed. Wright, Bristol, 1986 : 273-283.
3. Haagensen CD, Bodian C, Haagensen DE, Jr. Breast carcinoma. Risk and detection. W.B. Saunders, Philadelphia, London, Toronto, Mexico City, Sydney, and Tokyo, 1981.

Abbreviations of Journal names should conform to those of the Index Medicus. Provide all authors’ names when there are five or fewer co-authors. If there are more than five co-authors, list only the first three and use et al. If the title of a paper is in a language other than English, French or German, it should be translated into English, and the original language should be indicated in parentheses; for example, (in Japanese). Authors are responsible for the bibliographic accuracy of all references. Every reference must be checked, both in the manuscript and in proof.

**Tables.** Tables should be typed on separate sheets, numbered in Arabic numerals, and must have a brief title.

### Illustrations

Photographs, charts, graphs and diagrams are termed figures and should be numbered consecutively in Arabic numerals. Line drawings and graphs should be professionally drawn and lettered ; freehand and typewritten lettering is unacceptable. Photomicrographs and other photographic images should be submitted individually as unmounted original black-and-white prints. If authors wish to have a group of photographs printed together in a single block (composite figure), one set of photographs may be mounted to show the preferred layout. Photographs must be sized to fit within one column width (8.5 × 7.5 cm or 8.5 × 11.5 cm) or if needed, across two columns (17 × 11.5 cm). The maximum plate area for composite figures is 17×23 cm including the legend printed at its foot. Inappropriately sized material will be cropped or reduced by the Editorial Office or by the publisher. On the back of each figure, lightly write in very soft pencil the figure number, an indication of the top edge, and the last name of the first author. The figure number should not be placed on the printed surface. Letters, symbols, and arrows applied to the surface of a photograph must be of professional quality and sufficiently large to allow easy recognition. Dry transfer lettering (such as Letraset) may be used. All legends should appear together on a separate page(s). Legends should be brief and specific. Indicate the staining method and magnification for each photomicrograph. Use of scale markers in the image is suggested for electron micrographs.

**Color Illustrations.** When color illustrations are preferred, submit original color transparencies as well as three sets of color prints. The color prints should be trimmed to eliminate unnecessary marginal detail.

### Borrowed Material

Illustrations and tables borrowed from other sources and verbatim quotations totaling 200 words or more must be fully identified as to original author and source. Written permission must be obtained from both the original author and the publisher. Letters granting such permission should be forwarded with the manuscript.

### Publication Fees

Printing fees will be charged to the author according to the length of the manuscript and number of illustrations. Color printing and redrafting of tables require an additional charge. Reprints are supplied at rates based on the number of pages in the printed article and the number of reprints ordered. A reprint order form will be sent to the corresponding author along with the proofs. The order should be returned with the corrected proofs.

Publication fees could be exempted by allowance of the editorial board.

### Submission Fees

Submission fees (¥5,000, preferably by a postal money order) should be included at the time of submission since January, 1999. Foreign authors are tentatively exempted from a submission fee.

